# The Causation of Sex

**Published:** 1909-05-22

**Authors:** E. Rumley Dawson

**Affiliations:** Broadstairs


### Editor's Letter-Box.

### "THE CAUSATION OF SEX."

*To the Editor of* The Hospital.

Sir,—You will, I feel sure, allow me to reply to the review which appeared in The Hospital of April 24 of my book "The Causation of Sex," the more so as I am therein stated to make use of an illogical argument. Your reviewer first complains of my remarks being "too reiterative." This is scarcely fair criticism, as in the Introduction, p. 4, I claim the indulgence of the reader for reiteration, for I point out that "emphasis requires repetition and hence I fear the narrative suffers thereby." It is certainly gratifying that your reviewer could find fault with little beyond that for which the writer had already apologised.

Your reviewer next states that I illogically claim that the right ovary produces boys *because* it is the larger ovary and more boys are born than girls. This is certainly not my argument. I prove, as I think, from cases of normal pregnancy boy in utero, corpus luteum in right ovary; of ovarian pregnancy boys developing in right ovary, and girl in left ovary; of tubal pregnancies male in right tube, corpus luteum in right ovary; also from birth of boys only when left ovary was proved to be congenitally absent; as well as from cases of unilateral ovariotomy—that male children are derived from ova from the right ovary, and similarly females from the left ovary only.

Then I point out what is often overlooked, namely the anatomical fact that the right ovary is larger than the left, and therefore capable of producing more ova; we thus get the explanation why more boys are born than girls, because from the larger or right ovary I have just proved only male ova are derived. Similarly, we see why twin boys far more often appear than twin girls, because the larger area of ovarian tissue on the right side is more likely to produce two ova at or about the same time than the smaller area of the left ovary would be. Your reviewer appears to be surprised that I have not explained that the larger R. ovary "is simply in accordance with the more general right-handedness of men and women." I should certainly deny that this is cause and effect. If your reviewer credits it perhaps he will explain why, in spite of the right-handedness of men, the left testicle is larger than the right; and, though most women are right-handed, why the left kidney is larger and heavier than the right.

*Re* polyspermy, there is not the slightest evidence (only analogy derived from the invertebrata) in favour of only one spermatozoon being in mankind, not only the fertilising agent, but having also within it sufficient male germinal plasma to convey to, or impart, the divers male or paternal hereditary variations, characteristics, and peculiarities, including shape of ears, nails, nose, etc., size of head, mouth, feet, etc., colour of hair, skin, eyes, etc., tendencies to various diseases, and a hundred and one other details, which in varying combinations account for the differences, mental, moral, and physical, seen when there are several children to the same two parents.

Yours truly,

Broadstairs. E. Rumley Dawson.

